# 气相色谱法同时测定医疗器械产品中环氧乙烷、2-氯乙醇和乙二醇残留

**DOI:** 10.3724/SP.J.1123.2024.01010

**Published:** 2024-11-08

**Authors:** Ruojin LIU, Baoyu LIU, Hui LI, Wenliang SHAO, Yi FENG

**Affiliations:** 河北省药品医疗器械检验研究院, 河北 石家庄 050227; Hebei Province Testing Institute for Drug and Medical Devices, Shijiazhuang 050227, China

**Keywords:** 医疗器械, 气相色谱法, 环氧乙烷, 2-氯乙醇, 乙二醇, medical devices, gas chromatography (GC), ethylene oxide (EO), 2-chloroethanol (ECH), ethylene glycol (EG)

## Abstract

基于气相色谱法建立了同时快速测定经环氧乙烷灭菌后医疗器械产品中环氧乙烷、2-氯乙醇和乙二醇残留量的分析方法。称取2.5 g样品,加入5 mL乙醇浸提介质,在40 ℃的浸提温度下对样品中残留物质进行浸提,浸提时间为4 h。采用液体进样方式,经DB-WAX毛细管色谱柱分离,使用氢火焰离子化检测器进行测定。升温条件为40 ℃保持5 min, 40 ℃/min升温至120 ℃保持5 min, 6 ℃/min升温至200 ℃保持2 min,载气为氮气,流速为3 mL/min,采用外标法定量。环氧乙烷、2-氯乙醇和乙二醇在其相应的范围内线性关系良好,相关系数均大于0.99,检出限为0.10~0.40 μg/g,定量限为0.30~1.20 μg/g,不同加标水平下的回收率为91.08%~116.08%,相对标准偏差(*n*=6)为0.56%~8.45%。该分析方法灵敏、快速、准确,可用于科学有效评价环氧乙烷灭菌的一次性使用医疗器械产品中环氧乙烷相关残留物质的风险。

## Abstract (en)

A gas chromatography-based method was developed for the simultaneous and rapid determination of ethylene oxide (EO), 2-chloroethanol (ECH), and ethylene glycol (EG) residues in medical devices after EO sterilization. A sample weighing 2.5 g was added with 5 mL of ethanol as the extraction medium, and the residual substances in the sample were extracted at 40 ℃ for 4 h. The samples were separated on a DB-WAX capillary column (30 m×0.53 mm×1.0 μm) and determined using a hydrogen flame ionization detector. The temperature was maintained at 40 ℃ for 5 min, increased to 120 ℃ at a rate of 40 ℃/min, held for 5 min, and then increased to 200 ℃ at a rate of 6 ℃/min, held for 2 min. The flow rate of the nitrogen gas was 3 mL/min. The split ratio was 5∶1. The inlet and detector temperatures were 200 and 300 ℃, respectively. The changes in the chromatographic peak areas over time (0.5-10 h) under different temperatures (20, 30, 40, and 50 ℃) were investigated, and the optimal extraction condition was determined to be 40 ℃ for 4 h. In the experiments, quantification was performed using an external standard method. EO, ECH, and EG exhibited good peak shapes and separation effects as well as good linearity within their respective ranges. The linear correlation coefficients for EO, ECH, and EG were greater than 0.99. The limits of detection (LODs) for EO, ECH, and EG were in the range of 0.10-0.40 μg/g, and the limits of quantification (LOQs) were in the range of 0.30-1.20 μg/g. The average recoveries under different spiked levels were in the range of 91.08%-116.08%, and the relative standard deviations (*n*=6) were in the range of 0.56%-8.45%. EO, ECH, and EG residues were found to exist at different levels in the medical devices tested. In particular, disposable infusion sets must be paid careful attention. ECH and EG were not detected in disposable sterile medical devices made of non-polyvinyl chloride materials, which may be due to the fact that the products themselves did not contain chloride ions, they were not exposed to chlorine-containing substances during their production, sterilization, storage, transportation, use, etc. This study established a method to detect EO residues in disposable medical devices, and has the advantages of simple operation, excellent specificity, accurate quantification, and good reproducibility. It can simultaneously detect three residual substances in medical devices while meeting the actual detection requirements for EO, ECH, and EG residues. The method can be used to scientifically and effectively evaluate the risk of EO residues in single-use medical devices sterilized with EO, and will be helpful for improving the quality of medical devices, ensuring the safety of device use, and providing a reference for regulatory supervision and testing.

环氧乙烷(ethylene oxide, EO)具有成本低、低温低湿以及穿透性强的优势,广泛应用于一次性无菌医疗器械的灭菌^[1⇓-3]^。但EO是一种中枢神经抑制剂,与皮肤接触后会迅速发生红肿,若长期接触,会造成神经衰弱综合征和植物神经功能紊乱^[4,5]^,美国国家卫生研究院已将EO归为“已知的人类致癌物”^[6]^。为保障医疗器械使用的安全性,对于环氧乙烷灭菌的产品,EO残留量是必检项目。

与此同时,医疗器械产品经EO灭菌后,在存储、运输、使用等环节,残留的EO还存在生成其他副产物的可能。一方面,若残留的EO发生水解,进而与产品本身或环境中的氯离子反应生成2-氯乙醇(ethylene chlorohydrin, ECH)^[7]^。ECH是一种刺激体表、具有急性毒性,并且可以通过皮肤快速吸收的易燃性液体^[8]^。ECH具有轻微的致突变性,存在对肺、肾、中枢神经系统和心血管系统造成损伤的风险^[8]^。另一方面,若残留的EO与水反应,则会生成乙二醇(ethylene glycol, EG)。虽然EG性质稳定,不易挥发且毒性较低,但EG的代谢产物为乙醛酸和草酸。二者对人体的毒性较高,乙醛酸会抑制三羧酸循环,草酸则能引起代谢性酸中毒和肾损伤^[9]^。

目前,针对EO、ECH和EG 3种物质的单独检测,多采用气相色谱法(gas chromatography, GC)^[10-11]^和气相色谱-质谱法(gas chromatography-mass spectrometry, GC-MS)^[12⇓⇓-15]^。例如,闫顺华等^[16]^建立了顶空-气相色谱法检测口罩、防护服等医疗防护用品中EO的残留量,其方法检出限可以达到0.1 mg/kg。付步芳等^[17]^建立了GC-MS分析ECH残留量,并对经皮经动脉血管成形术(PTA)球囊扩张导管的ECH残留量水平进行了安全性评价。范晨亮等^[18]^建立了测定工业用EG纯度及其中有机杂质的气相色谱方法,可满足工业化分析乙二醇纯度和杂质的基本要求,在乙二醇生产控制、产品检测等方面具有良好的应用前景。

在同时检测方面,现有的研究文献多集中于EO和ECH二者的共同测定,且多集中在食品和药品领域^[19⇓⇓-22]^。例如,周静等^[23]^采用顶空-气相色谱-质谱技术,实现了对乳及乳制品(液态乳、发酵乳、婴幼儿配方乳粉、冰淇淋)中EO和ECH的准确定性和定量。周云婷等^[24]^采用气相色谱法建立了同时测定EO和ECH残留量的方法,可用于明胶空心胶囊中EO和ECH的同时控制。

目前尚未检索到同时测定医疗器械产品中EO、ECH和EG的相关报道。与此同时,现行标准尚无适用于医疗器械产品中EO、ECH和EG的同时检测。因此如何实现3种物质的快速有效检测对医疗器械的监管检测意义重大。

本文基于气相色谱法建立了同时快速测定EO、ECH和EG残留量的分析方法,并采用建立的方法对典型一次性使用无菌医疗器械产品进行了3种物质残留量的检测,可为医疗器械相关产品中环氧乙烷残留物质的判断提供技术评价依据,为监管部门的监督检测提供参考。

## 1 实验部分

### 1.1 仪器、试剂与材料

8890B气相色谱仪(美国Agilent公司),配有HA-300氢空一体机(北京中惠分析技术研究所)和7693A液体进样器(美国Agilent公司); Milli-Q超纯水机(美国Millipore公司); MS104TS电子天平(美国Mettler Toledo公司); SHP080生化培养箱(上海精宏实验设备有限公司)。

EO标准品(质量浓度为50000 mg/L)购自上海安谱璀世标准技术服务有限公司;ECH标准品(100 mg/支)购自美国Panphy Chemicals公司;EG标准品(色谱纯)购自上海阿拉丁试剂有限公司;乙醇(色谱纯)、丙酮(分析纯)均购自天津市科密欧化学试剂有限公司,乙腈(色谱纯)购自上海麦克林生化科技股份有限公司。

本试验医疗器械产品检测阶段涉及以下产品,均经EO灭菌。按产品主要实验部位材质分类如下:聚氯乙烯材质,一次性使用鼻氧管、一次性使用湿化鼻氧管、一次性使用吸氧管、一次性使用湿化吸氧管、一次性使用聚氯乙烯导尿管、一次性使用无菌组织冲洗器、一次性使用输液器、一次性使用输液器(带针);高分子硅橡胶材质,一次性使用子宫测量管;聚丙烯材质,一次性医用包布、一次性使用无菌包布;聚乙烯材质,一次性使用超低密度聚乙烯输液器;硅胶材质,一次性使用硅胶导尿管;热塑性弹性体材质,一次性使用输液器;聚苯乙烯材质,一次性使用无菌阴道扩张器。

### 1.2 标准溶液的配制

1000 μg/mL混合贮备液配制:精密量取2.0 mL 的EO标准品,精密称重100.0 mg ECH标准品和100.0 mg EG,置于100 mL容量瓶中(已加约80 mL乙醇),用乙醇稀释至刻度。

将1000 μg/mL的混合贮备液用乙醇逐级稀释,得到质量浓度为0.15、0.30、0.60、1.20、1.50、3.00、6.00、10.00 μg/mL的混合标准溶液。分别取15种未经环氧乙烷灭菌的产品样品2.5 g,依次加入各浓度混合标准溶液5.0 mL,于40 ℃下放置4 h,将样品与液体分离,过滤后取1.0 mL溶液于样品瓶中,立即密封,得到含量分别为0.30、0.60、1.20、2.40、3.00、6.00、12.00、20.00 μg/g的EO、ECH与EG基质校对标准溶液。

### 1.3 样品浸提液制备

取包装完整的一次性使用无菌医疗器械产品,剪成约5 mm长碎块;其中一次性医用包布、一次性使用无菌包布和一次性使用无菌阴道扩张器剪成约0.5 cm^2^碎块。取样品2.5 g于25 mL具塞玻璃比色管中,加入5.0 mL乙醇后密封,于40 ℃下浸提4 h,将样品与液体分离,过滤后取1.0 mL浸提液于样品瓶中,密封。平行制备2份。

### 1.4 气相色谱条件

色谱柱:DB-WAX(30 m×0.53 mm×1.0 μm);柱温升温程序:40 ℃保持5 min, 40 ℃/min升温至120 ℃,保持5 min, 6 ℃/min升温至200 ℃,保持2 min;进样口温度:200 ℃;检测器温度:300 ℃;载气:氮气,流速:3 mL/min;分流比:5∶1。

## 2 结果与分析

### 2.1 进样方式的选择

EO的沸点低、易挥发,适用于顶空进样方式进行测定^[12]^。ECH具有一定的挥发性,而EG沸点高不易挥发,若采用顶空进样的方式,容易因为平衡温度过高而使顶空瓶内气压过大导致破裂。因此,液体直接进样的方式更适用于三者的同时检测。

### 2.2 气相色谱柱的选择

目前,关于环氧乙烷残留物质的测定,文献中普遍选用的色谱柱有强极性柱HP-INNOWAX^[14,15]^、DB-WAX^[9]^和中等极性柱DB-624^[10,22,24]^。分别考察了HP-INNOWAX(60 m×0.53 mm×1.0 μm)、DB-WAX(30 m×0.53 mm×1.0 μm)和DB-624(30 m×0.53 mm×3.0 μm)3种色谱柱对残留物质的分离效果。结果发现,3种物质在DB-WAX色谱柱上响应值较高,峰形良好,分析时间较短且分离效果最好(见图1)。因此,选择DB-WAX对一次性使用无菌医疗器械产品进行EO、ECH与EG残留量的测定。

**图1:**
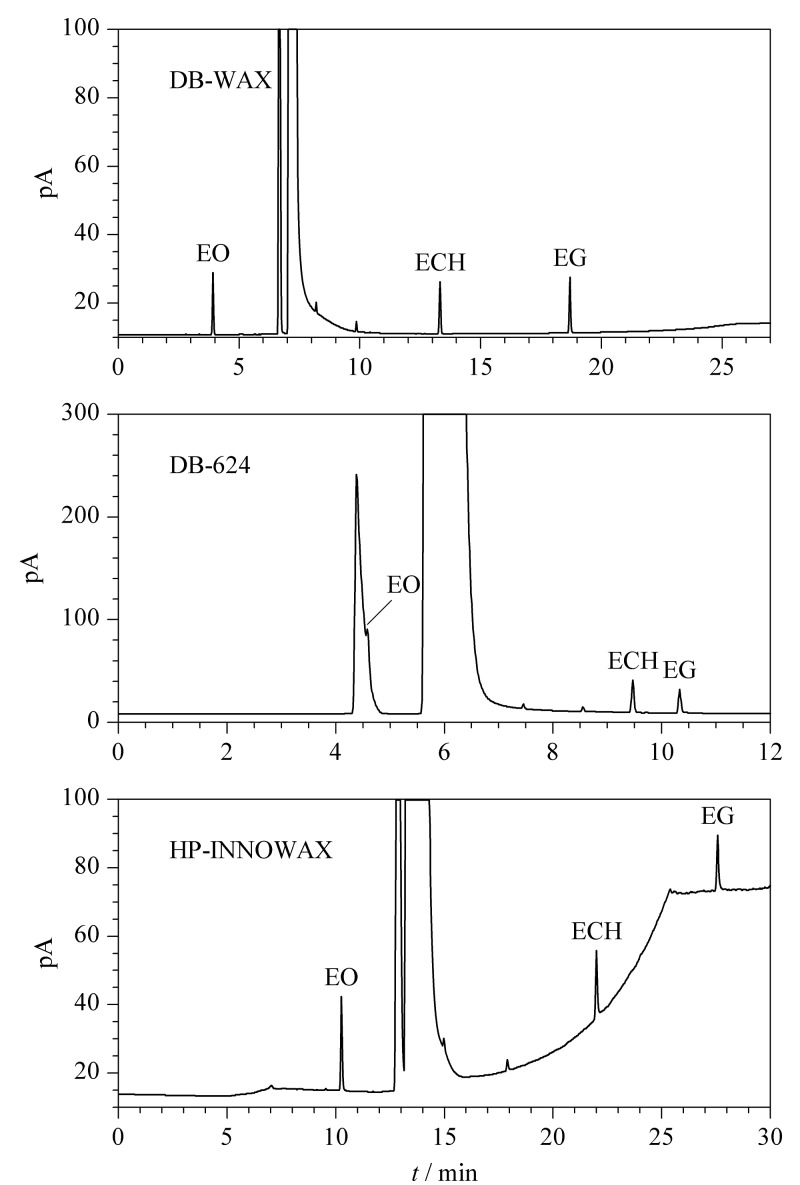
EO、ECH与EG混合标准溶液的气相色谱图

### 2.3 浸提条件的选择

分别考察了浸提介质、浸提温度、浸提时间和取样量对检测结果的影响。

#### 2.3.1 浸提介质的确定

目前文献中测定EO、ECH与EG的浸提介质有水、乙醇、丙酮和乙腈等^[9,11,20,24,25]^。考虑到ECH在水中不稳定^[26]^,且EO遇水易生成EG,干扰EO和EG的真实含量;同时,当水作为浸提介质直接进样时,可能会出现衬管过载、色谱柱分离度和重现性差等问题,故水作为浸提介质不适用于这3种物质的测定。

取经验证含EO、ECH和EG的一次性使用输液器样品2.5 g,依次以乙醇、丙酮和乙腈作为浸提介质,按1.3节下操作制备样品浸提液并测定。结果发现,丙酮由于沸点较低,挥发过快,无法实现产品浸提;且丙酮属于易制毒试剂,故不选用。经乙醇和乙腈浸提的测定结果见图2,相比于乙醇而言,乙腈作为浸提介质时浸出杂质过多,容易干扰目标物质测定。此外,乙醇具有成本低、毒性小的优势,故选用乙醇作为浸提介质。

**图2:**
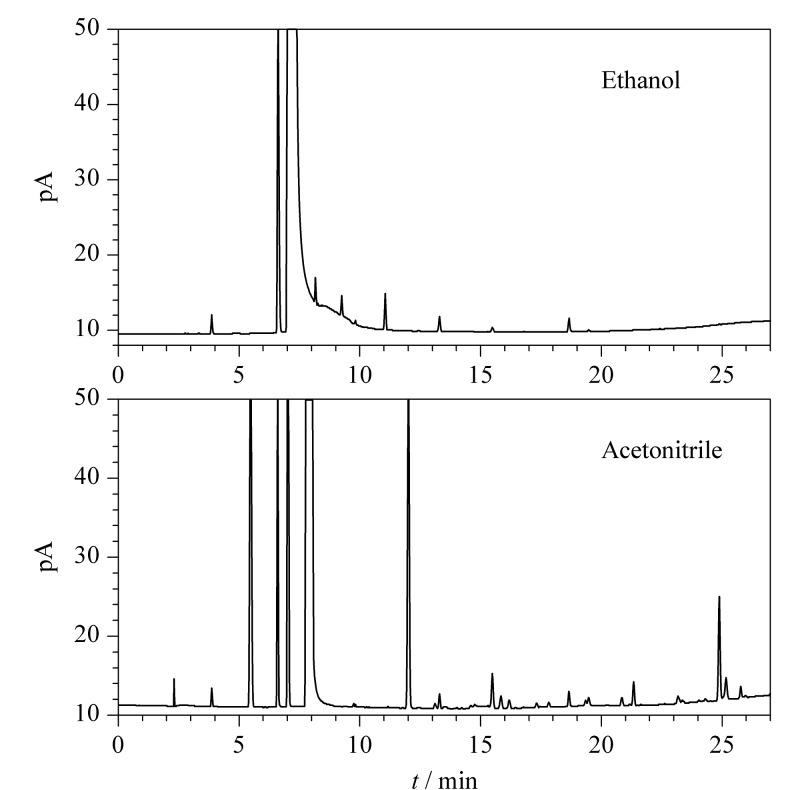
经灭菌样品在不同浸提介质中的气相色谱图

#### 2.3.2 浸提温度和浸提时间的确定

考察了浸提温度和浸提时间对3种残留物质浸提量的影响(见图3)。取经验证含EO、ECH与EG的一次性使用输液器,分别于20、30、40和50 ℃温度下浸提0.5、1、2、3、4、5、7、9、10 h。

**图3:**
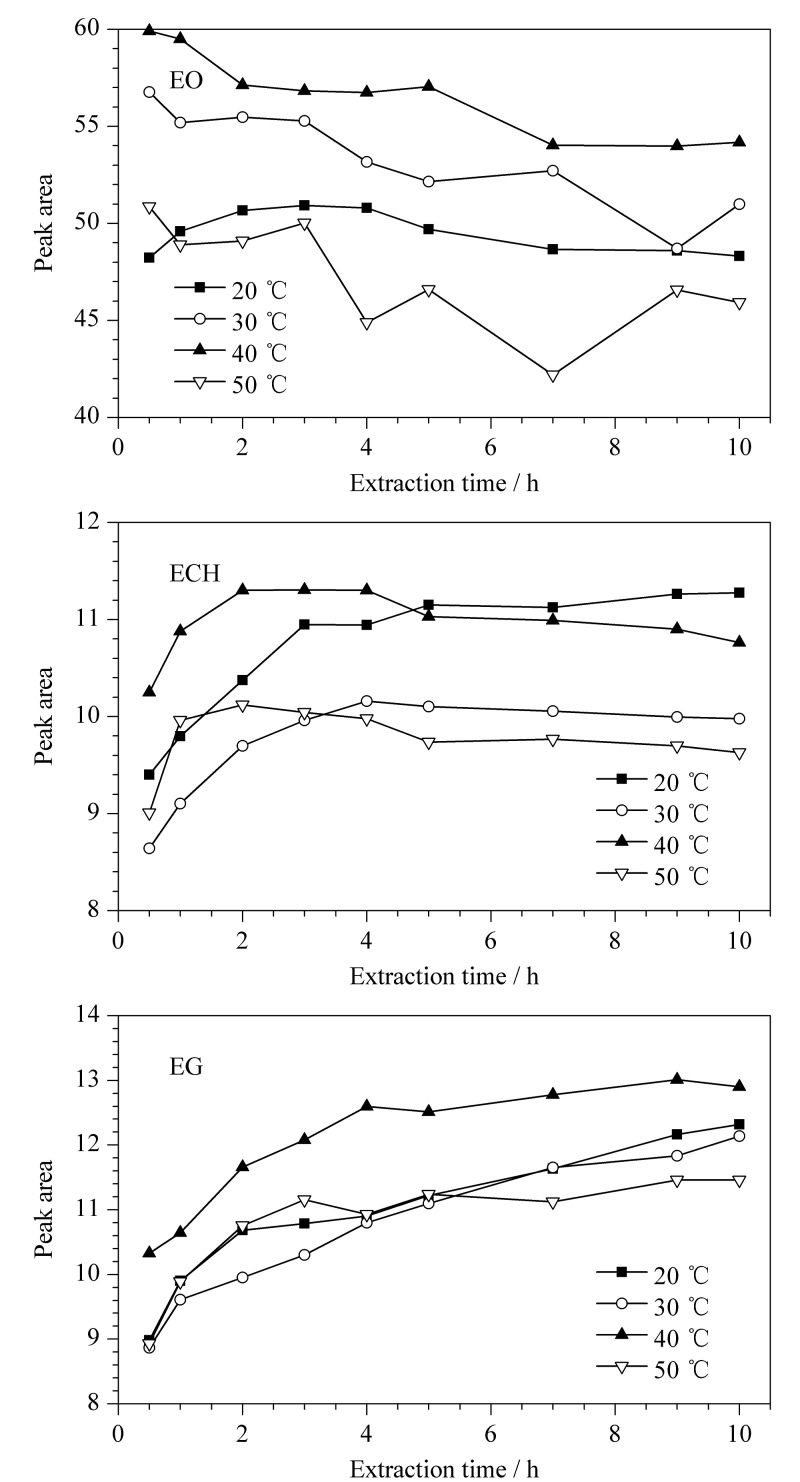
不同温度下EO、ECH与EG浸提量随时间的变化

由图3可知,沸点差异较大的3种目标物质所展现出的浸提规律具有明显的差异。首先,针对低沸点的EO而言,随着浸提时间的延长,各温度条件下浸提量整体呈现下降趋势,说明对于沸点低的EO,不宜进行长时间的浸提。在20~40 ℃的浸提温度区间内,EO整体浸提量:40 ℃>30 ℃>20 ℃,表明一定范围内温度的提高有助于促进EO的浸提效果。但当温度在50 ℃时,较高的温度会促使EO从浸提介质乙醇中逸出,浸提量反而降低。

其次,对于沸点介于EO和EG之间的ECH而言,随着浸提时间越长,其峰面积先增大后趋于稳定。另外,在浸提开始阶段,浸提温度越高,ECH峰面积的增大速率越快,说明温度有助于提升ECH的浸提速率。当浸提温度为40 ℃,浸提时间为4 h时,ECH的浸提量最大。

对于沸点高的EG而言,各温度条件下,其峰面积随浸提时间延长均呈现逐渐增大的趋势,由于其沸点较高不易挥发,浸提量随浸提时间延长而增大。40 ℃下EG整体浸提量均高于其余温度条件,4 h后其浸提速率放缓。3种物质的浸提规律并非仅受温度或时间单因素的影响,物质之间的相互作用有待进一步探究。

为同时实现3种目标物质较好的浸提效果,最终选择40 ℃下平衡4 h。

#### 2.3.3 样品质量的确定

取经验证含EO、ECH与EG的一次性使用输液器进行分析,分别向具塞玻璃比色管中加入0.5、1.0、1.5、2.0、2.5、3.0、4.0、5.0 g样品,按1.3节操作平行制备浸提液3份,使用1.4节的仪器条件进行测定。以样品质量为横坐标,峰面积为纵坐标绘制曲线图(见图4)。

**图4:**
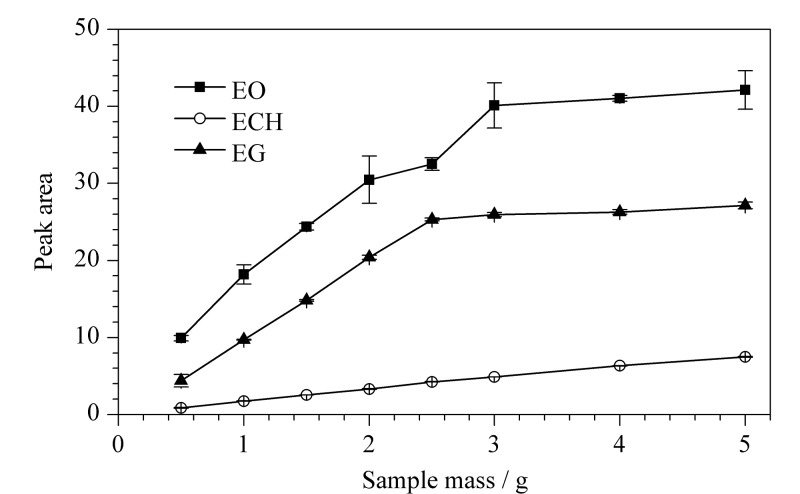
取样量对EO、ECH与EG峰面积的影响(*n*=3)

结果发现,EO、ECH与EG峰面积整体趋势是随着样品质量的增大而增大,但只有ECH的峰面积与样品质量呈线性增加关系,而对EO和EG而言,随样品质量增大,峰面积增加速度先快后慢,分别在样品质量为3.0 g和2.5 g后峰面积缓慢增加。这表明在3种物质共存的体系中,浸提量与样品质量之间并非简单的线性关系,物质之间存在相互影响。但考虑到当样品质量大于2.5 g时,样品所占的体积较大,乙醇不能完全浸湿样品表面,不能代表样品中3种物质的真实残留量,因此最终确定样品质量为2.5 g。

### 2.4 线性关系、检出限和定量限

将1.2节配制好的基质校对标准溶液按1.4节仪器条件进行测定,以保留时间定性,分别以标准物质的含量为横坐标(*X*, μg/g)、峰面积(*Y*)为纵坐标绘制标准曲线,得到不同产品的EO、ECH与EG的线性方程和相关系数(*r*)。以3倍和10倍信噪比分别确定各产品的检出限和定量限^[21]^,相关数据见表1。

**表1:** EO、ECH与EG在不同产品中的线性范围、线性方程、相关系数、检出限和定量限

Product name	Compound	Linear range/(μg/g)	Linear equation	*r*	LOD/(μg/g)	LOQ/(μg/g)
Disposable nasal oxygen tube	EO	0.30-20.00	*Y*=0.3174*X*+0.5695	0.9992	0.10	0.30
	ECH	0.60-20.00	*Y*=0.3523*X*+0.0248	0.9999	0.18	0.56
	EG	1.20-20.00	*Y*=0.3406*X*+0.0860	0.9991	0.36	1.10
Disposable humidifying nasal	EO	0.30-20.00	*Y*=0.3025*X*+0.6103	0.9992	0.10	0.30
oxygen tube	ECH	0.60-20.00	*Y*=0.3468*X*+0.0425	0.9998	0.18	0.56
	EG	1.20-20.00	*Y*=0.3591*X*+0.0137	0.9990	0.36	1.10
Disposable use of oxygen tube	EO	0.30-20.00	*Y*=0.3056*X*+0.6045	0.9965	0.10	0.30
	ECH	0.60-20.00	*Y*=0.3581*X*+0.0171	0.9995	0.18	0.56
	EG	1.20-20.00	*Y*=0.3754*X*+0.0244	0.9940	0.36	1.10
Disposable humidification	EO	0.30-20.00	*Y*=0.3034*X*+0.6029	0.9991	0.10	0.30
oxygen tube	ECH	0.60-20.00	*Y*=0.3583*X*+0.0160	0.9996	0.18	0.56
	EG	1.20-20.00	*Y*=0.3519*X*+0.0390	0.9956	0.36	1.10
Disposable PVC catheter	EO	0.30-20.00	*Y*=0.3738*X*+0.4318	0.9999	0.10	0.30
	ECH	0.60-20.00	*Y*=0.3960*X*+0.0606	0.9997	0.20	0.60
	EG	1.20-20.00	*Y*=0.4080*X*+0.1004	0.9998	0.38	1.20
Disposable sterile tissue irrigator	EO	0.30-20.00	*Y*=0.3553*X*+0.5800	0.9990	0.10	0.30
	ECH	0.60-20.00	*Y*=0.4050*X*-0.0084	0.9999	0.20	0.60
	EG	1.20-20.00	*Y*=0.4076*X*+0.1305	0.9998	0.38	1.20
Disposable infusion set	EO	0.30-20.00	*Y*=0.3130*X*+0.9297	0.9990	0.10	0.30
	ECH	0.60-20.00	*Y*=0.3744*X*+0.2619	0.9995	0.20	0.60
	EG	1.20-20.00	*Y*=0.4576*X*+0.4137	0.9944	0.38	1.20
Disposable infusion set with needle	EO	0.30-20.00	*Y*=0.3106*X*+0.9626	0.9992	0.10	0.30
	ECH	0.60-20.00	*Y*=0.3718*X*+0.2701	0.9983	0.20	0.60
	EG	1.20-20.00	*Y*=0.4437*X*+0.6147	0.9955	0.38	1.20
Disposable uterine measuring tube	EO	0.30-20.00	*Y*=0.3799*X*+0.1625	0.9974	0.10	0.30
	ECH	0.60-20.00	*Y*=0.4215*X*-0.1448	1.0000	0.20	0.60
	EG	1.20-20.00	*Y*=0.3772*X*+0.1371	0.9960	0.38	1.20
Disposable medical bag cloth	EO	0.30-20.00	*Y*=0.4014*X*+0.7481	0.9969	0.12	0.32
	ECH	0.60-20.00	*Y*=0.4186*X*+0.0308	0.9998	0.22	0.62
	EG	1.20-20.00	*Y*=0.3625*X*+0.2837	0.9992	0.40	1.20
Disposable sterile wrapping cloth	EO	0.30-20.00	*Y*=0.3839*X*+0.8396	0.9998	0.12	0.32
	ECH	0.60-20.00	*Y*=0.4196*X*+0.0252	1.0000	0.22	0.62
	EG	1.20-20.00	*Y*=0.3714*X*+0.1560	0.9987	0.40	1.20
Disposable ultra low density	EO	0.30-20.00	*Y*=0.3486*X*+0.6658	0.9998	0.10	0.30
polyethylene infusion set	ECH	0.60-20.00	*Y*=0.3809*X*+0.1973	1.0000	0.20	0.60
	EG	1.20-20.00	*Y*=0.4078*X*+0.9092	0.9997	0.38	1.20
Disposable silicone catheter	EO	0.30-20.00	*Y*=0.3475*X*+0.5848	0.9998	0.10	0.30
	ECH	0.60-20.00	*Y*=0.4125*X*+0.0234	0.9998	0.22	0.60
	EG	1.20-20.00	*Y*=0.3677*X*+0.3763	0.9998	0.38	1.20
Disposable infusion set	EO	0.30-20.00	*Y*=0.3382*X*+0.7976	0.9998	0.10	0.30
(thermoplastic elastomers)	ECH	0.60-20.00	*Y*=0.3780*X*+0.2400	1.0000	0.20	0.60
	EG	1.20-20.00	*Y*=0.4100*X*+0.7449	0.9995	0.38	1.20
Disposable use of sterile	EO	0.30-20.00	*Y*=0.3469*X*+0.5930	0.9998	0.10	0.30
vaginal dilator	ECH	0.60-20.00	*Y*=0.3914*X*+0.0757	0.9999	0.18	0.60
	EG	1.20-20.00	*Y*=0.3692*X*+0.3705	0.9999	0.36	1.10

*Y*: peak area; *X*: content, μg/g. PVC: polyvinyl chloride.

由表1可以看出,3种物质在线性范围内线性关系良好,在不同样品中的检出限为0.10~0.40 μg/g,定量限为0.30~1.20 μg/g。该方法线性范围宽,灵敏度高。

### 2.5 回收率和精密度

分别取15种未经环氧乙烷灭菌的产品样品2.5 g,在1.20、2.40、12.00 μg/g 3个加标水平下加入标准溶液5.0 mL,于40 ℃下放置4 h,将样品与液体分离,过滤后取1.0 mL溶液于样品瓶中,每个添加水平重复测定6次,通过加标回收试验考察方法的准确度,结果见表2。EO、ECH与EG 3种物质的平均回收率为91.08%~116.08%, RSD为0.56%~8.45%。该方法准确度及精密度良好,满足检测要求。

**表2:** EO、ECH与EG在不同基质中的回收率和相对标准偏差(*n*=6)

Product name	Added/ (μg/g)	EO			ECH			EG	
		Recovery/%	RSD/%		Recovery/%	RSD/%		Recovery/%	RSD/%
Disposable nasal oxygen tube	1.20	109.61	8.03		101.48	4.78		114.26	7.85
	2.40	105.74	4.11		103.43	3.00		98.11	2.60
	12.00	113.05	2.81		100.46	3.62		103.15	2.30
Disposable humidifying nasal oxygen tube	1.20	96.84	5.52		104.69	4.06		100.82	6.03
	2.40	100.28	4.04		97.24	3.35		103.61	3.19
	12.00	107.01	1.25		110.05	0.86		106.33	2.01
Disposable use of oxygen tube	1.20	99.85	5.70		100.27	6.40		114.71	3.01
	2.40	107.03	4.01		107.91	2.00		95.93	2.54
	12.00	102.87	2.66		98.10	1.65		101.41	1.99
Disposable humidification oxygen tube	1.20	104.64	6.16		107.72	3.99		110.87	3.20
	2.40	107.51	3.39		108.19	1.69		102.64	4.69
	12.00	99.29	1.66		97.56	0.91		106.54	3.43
Disposable PVC catheter	1.20	106.05	6.59		109.55	6.69		106.98	4.63
	2.40	104.82	4.63		109.49	4.14		104.23	3.65
	12.00	100.01	2.35		101.16	1.94		103.59	0.68
Disposable sterile tissue irrigator	1.20	108.05	5.16		107.56	4.42		100.25	6.69
	2.40	97.06	2.62		91.26	4.02		96.86	4.00
	12.00	101.40	0.92		100.78	1.29		103.16	1.94
Disposable infusion set	1.20	95.75	4.71		102.33	5.44		114.60	3.72
	2.40	108.20	5.61		99.82	3.45		105.85	2.79
	12.00	106.03	1.40		100.85	1.68		97.17	1.72
Disposable infusion set with needle	1.20	94.55	7.75		102.71	4.20		99.22	6.44
	2.40	98.07	6.12		104.42	5.16		106.20	2.86
	12.00	100.88	3.64		99.33	0.59		108.28	1.46
Disposable uterine measuring tube	1.20	99.22	6.44		116.08	2.24		100.90	7.18
	2.40	106.20	2.86		106.66	2.52		104.32	4.54
	12.00	108.28	1.46		99.58	2.12		101.91	1.75
Disposable medical bag cloth	1.20	91.08	5.40		98.55	2.88		97.08	4.76
	2.40	93.74	4.29		99.87	4.35		96.61	5.06
	12.00	98.55	0.56		99.50	2.10		95.13	1.91
Disposable sterile wrapping cloth	1.20	92.63	8.45		96.45	4.07		102.95	7.61
	2.40	99.46	3.38		106.94	2.40		99.45	3.51
	12.00	100.27	1.40		103.11	1.69		96.13	3.48
Disposable ultra low density polyethylene	1.20	97.98	3.28		110.49	6.37		102.51	6.10
infusion set	2.40	105.41	4.71		103.87	5.26		100.60	4.34
	12.00	104.36	3.33		97.54	2.97		97.74	2.14
Disposable silicone catheter	1.20	112.00	5.84		110.05	3.22		101.50	4.96
	2.40	106.40	3.13		97.13	2.38		102.37	2.77
	12.00	99.63	1.49		102.87	1.82		104.29	1.99
Disposable infusion set (thermoplastic	1.20	110.08	5.04		104.17	4.89		100.87	3.53
elastomers)	2.40	105.76	2.71		98.60	3.87		107.33	2.46
	12.00	106.18	1.27		100.01	1.60		101.15	1.52
Disposable use of sterile vaginal dilator	1.20	91.69	3.54		103.15	4.77		100.71	3.25
	2.40	106.22	2.31		106.88	2.22		106.22	4.02
	12.00	97.14	1.08		99.96	0.65		102.17	1.95

### 2.6 医疗器械产品检测

选取经环氧乙烷灭菌的15种一次性使用医疗器械产品进行测定,结果见表3。共5个样品检出EO,为一次性使用鼻氧管、一次性使用无菌组织冲洗器、一次性使用输液器(带针)、一次性使用无菌包布和一次性使用医用包布,以上样品的EO残留量均小于10 μg/g,但不同样品的EO残留量相差较大,这可能是由于样品间材质差异造成的,不同材质对环氧乙烷的吸收、保持和释放能力有显著差异^[8]^。EO残留量的差异进而会影响ECH与EG的残留量。其次,H厂家的一次性使用输液器(带针)同时检出了3种残留物质,而G厂家的一次性使用输液器中均未检测到目标物质,表明不同厂家之间该类产品存在差异,应着重关注该类产品环氧乙烷相关残留物质的检测,降低安全风险。此外,非聚氯乙烯材质的样品均未检出ECH,这可能是由于样品原材料不含氯离子,同时在生产规范的情况下,其他途径未引入氯离子^[17]^。

**表3:** 医疗器械产品中3种环氧乙烷相关残留物质的测定结果

Manufacturer code	Product name	Main material	Contents/(μg/g)		
			EO	ECH	EG
A	disposable nasal oxygen tube	polyvinyl chloride	2.10	/	/
B	disposable humidifying nasal oxygen tube	polyvinyl chloride	/	/	/
C	disposable use of oxygen tube	polyvinyl chloride	/	/	/
D	disposable humidification oxygen tube	polyvinyl chloride	/	/	/
E	disposable PVC catheter	polyvinyl chloride	/	/	/
F	disposable sterile tissue irrigator	polyvinyl chloride	0.56	/	/
G	disposable infusion set	polyvinyl chloride	/	/	/
H	disposable infusion set with needle	polyvinyl chloride	0.84	1.28	1.54
I	disposable uterine measuring tube	polymer silicone rubber	/	/	/
J	disposable medical bag cloth	polypropylene	0.76	/	/
K	disposable sterile wrapping cloth	polypropylene	0.64	/	/
L	ultra low density polyethylene infusion set	polythene	/	/	/
M	disposable silicone catheter	silica gel	/	/	/
N	disposable infusion set	thermoplastic elastomer	/	/	/
O	disposable use of sterile vaginal dilators	polystyrene	/	/	/

/: not detected.

## 3 结论

本文基于气相色谱法建立了同时快速测定环氧乙烷灭菌的医疗器械产品中EO、ECH和EG残留量的分析方法,并利用该方法对经环氧乙烷灭菌的一次性使用输液器和一次性使用鼻氧管等共15批次的一次性使用医疗器械产品进行了测定。检出EO、ECH与EG存在不同程度的残留。该方法操作简单,定量准确,重复性好,完全可满足实际EO、ECH与EG残留量的检测需求,有助于科学有效地评价一次性使用无菌医疗器械产品中环氧乙烷残留物质的风险,对于提高医疗器械质量、保证器械使用安全十分必要,为监管部门的监督检测提供参考。

## References

[1] Yang S L, Zheng W W, Zhang W C, et al. China Medical Device Information, 2021, 27(5): 6

[2] Liu B D, He W Z, Chen M. Chinese Journal of Medical Instrumentation, 2020, 44(5): 443 10.3969/j.issn.1671-7104.2020.05.015 33047571

[3] Jildeh Z B, Wagner P H, Schoning M J. Phys Status Solidi A, 2021. DOI: 10.1002/pssa.202000732

[4] Liu C M, Shang B, Tian D. Chinese Journal of Industrial Medicine, 2021, 34(4): 316

[5] Chen L, Wei X, Chaves B D, et al. Food Microbiol, 2021, 94: 103656 33279081 10.1016/j.fm.2020.103656

[6] National Toxicology Program (NTP). Chemical Information Review Document for Ethylene Oxide. (2016-11-03) [2021-03-04]. http://ifc.helib.net:80/rwt/331/https/N34HALUPNFTXR63PN3VXRLUHN75A/ntp/roc/content/profiles/ethyleneoxide.pdfhttp://ifc.helib.net:80/rwt/331/https/N34HALUPNFTXR63PN3VXRLUHN75A/ntp/roc/content/profiles/ethyleneoxide.pdf

[7] YY/T 1690-2020

[8] GB/T 16886.7-2015

[9] Qiu Y X, Chen T T, Chen Y, et al. Hubei Agricultural Sciences, 2021, 60(20): 145

[10] Cheng L, Guo W X, Wang D D, et al. Analysis and Testing Technology and Instruments, 2023, 29(3): 299

[11] Gao G H, Wang X L, Dong Y, et al. Journal of Shenyang Pharmaceutical University, 2010, 27(5): 385

[12] Lei C N, Wang B, Liu A J, et al. Journal of Analytical Science, 2021, 37(1): 129

[13] Wu Z X, Fu B F, Wang S H, et al. Physical Testing and Chemical Analysis Part B: Chemical Analysis, 2015, 51(12): 1644

[14] Zhong Z H, Liu H Y, Wang P, et al. Chinese Journal of Analysis Laboratory, 2018, 37(9): 1071

[15] Chen F L, Hu J B, Pan Y S, et al. Shandong Chemical Industry, 2022, 51(3): 110

[16] Yan S H, Luo M, Zhu Y Z, et al. Chemical Analysis and Meterage, 2021, 30(3): 56

[17] Fu B F, Cheng D S, Wang C R. Beijing Biomedical Engineering, 2018, 37(1): 62

[18] Fan C L, Zhang Y H, Wang C, et al. Chinese Journal of Chromatography, 2019, 37(1): 116 10.3724/SP.J.1123.2018.08030 30693718

[19] Wang S, Jiang X M, He D P, et al. Journal of Grain and Oils, 2023, 36(9): 145

[20] Gong F, Hao L H, Li Z Z, et al. Journal of Food Safety and Quality, 2021, 12(24): 9349

[21] Jiang F, Wu W Q, Li X, et al. Food Science and Technology, 2021, 46(7): 295

[22] Lin H S, Huang Y F, Yue W H. Capital Medicine, 2014, 21(22): 102

[23] Zhou J, Xu H B, Zhou Z L, et al. Dairy Science and Technology, 2023, 46(1): 24

[24] Zhou Y T, Yang H P, Qian J, et al. Chemical Engineering Management, 2022(13): 47

[25] Milton O. J Assoc Off Agric Chem, 2020, 26(1): 99

[26] Fu B F, Feng X M. Chinese Journal of Pharmacovigilance, 2009, 6(6): 332

